# Mmu_circ_0000037 inhibits the progression of acute pancreatitis by miR‐92a‐3p/Pias1 axis

**DOI:** 10.1002/iid3.819

**Published:** 2023-04-12

**Authors:** Hua Chen, Jun Tu, Lei He, Ning Gao, Weiqiang Yang

**Affiliations:** ^1^ Department of Gastroenterology Fengxian District Central Hospital Shanghai China; ^2^ Department of General Internal Medicine, Ping An Health Internet Shanghai Branch Shanghai China; ^3^ Department of General Surgery Jiading District Central Hospital Shanghai China

**Keywords:** acute pancreatitis, inflammatory injury, miR‐92a‐3p, mmu_circ_0000037, Pias1

## Abstract

**Background:**

Acute pancreatitis (AP) is an inflammatory disease with high mortality. Previous study has suggested that circular RNAs are dysregulated and involved in the regulation of inflammatory responses in AP. This study aimed to investigate the function and regulatory mechanism underlying mmu_circ_0000037 in caerulein‐induced AP cellular model.

**Methods:**

Caerulein‐treated MPC‐83 cells were used as an in vitro cellular model for AP. The expression levels of mmu_circ_0000037, microRNA (miR)‐92a‐3p, and protein inhibitor of activated STAT1 (Pias1) were detected by quantitative real‐time polymerase chain reaction. Cell viability, amylase activity, apoptosis, and inflammatory response were detected by 3‐(4,5‐Dimethylthiazol‐2‐yl)‐2,5‐diphenyltetrazolium bromide, Amylase Assay Kit, flow cytometry, and enzyme‐linked immunosorbent assays. The protein level was quantified by western blot analysis. The target interaction between miR‐92a‐3p and mmu_circ_0000037 or Pias1 were predicted by StarbaseV3.0 and validated by dual‐luciferase reporter assay and RNA immunoprecipitation assay.

**Results:**

Mmu_circ_0000037 and Pias1 levels were decreased, whereas miR‐92a‐3p expression was elevated in caerulein‐induced MPC‐83 cells. Overexpression of mmu_circ_0000037 protected MPC‐83 cells from caerulein‐induced the decrease of cell viability, as well as the promotion of amylase activity, apoptosis and inflammation. MiR‐92a‐3p was targeted by mmu_circ_0000037, and miR‐92a‐3p overexpression rescued the effect of mmu_circ_0000037 on caerulein‐induced MPC‐83 cell injury. Pias1 was confirmed as a target of miR‐92a‐3p and mmu_circ_0000037 regulated the expression of Pias1 by sponging miR‐92a‐3p.

**Conclusion:**

Mmu_circ_0000037 relieves caerulein‐induced inflammatory injury in MPC‐83 cells by targeting miR‐92a‐3p/Pias1 axis, providing a theoretical basis for the treatment of AP.

## Graphical abstract

Mmu_circ_0000037 regulated cell apoptosis and inflammatory response by sponging miR‐92a‐3p to deregulate the regulation on miR‐92a‐3p target Pias1.

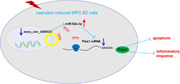

## 1 INTRODUCTION

Acute pancreatitis (AP) is a serious inflammatory disease with rising incidence worldwide.^1^ Cell death and inflammatory response are the classic pathologic features of AP.^2^ With efforts, great progress have been gained on understanding the pathogenesis and management of AP.^3^ However, the effective strategies remain limited for treatment of AP. Consequently, it is necessary to ascertain promising target for improving outcomes of patients with AP.

Circular RNAs (circRNAs) have been identified as vital elements in gene regulation under multiple physiological and pathological conditions.^4^ CircRNAs are formed by reverse splicing of RNA and have a covalent closed‐loop structure with high stability.^5^ There is emerging evidence that abnormal expression of circRNAs participate in the regulation of inflammation infiltration and multiple diseases complication, including AP.^6^, ^7^, ^8^ Thus, circRNAs have been deemed as perfect biomarkers for multiple diseases, including diagnosis, prognosis, and monitoring of treatment responses. For example, circRNA zinc finger protein 644 regulated the pathogenesis of AP by sponging miR‐21‐3p.^9^ In addition, next‐generation RNA sequencing identified several differentially expressed circRNAs and mmu_circ_0000037 was found to be significantly downregulated in the pancreatic tissues of three severe AP (SAP) mice.^9^ However, the function and regulatory mechanism underlying mmu_circ_0000037 have not been clarified.

On the basis of competing endogenous RNA (ceRNA) theory, circRNAs can function as microRNA (miRNA) sponge to regulate the stability and translation of target messenger RNAs (mRNAs).^10^ miRNAs are a form of noncoding RNAs that perform impressive roles in diseases by acting as potential targets of diagnosis and treatment.^11^ Moreover, many miRNAs have been regarded as key diagnostic and prognostic targets for AP.^12^ For example, increased miR‐551b‐5p had been reported to predict poor outcome of AP patients and was associated with inflammatory response.^13^ Moreover, miR‐148a could suppress inflammatory response and autophagy in caerulein‐induced AP model.^14^ High expression of miR‐92a‐3p was detected in AP rats and miR‐92a‐3p knockdown relieved AP pathological course.^15^ In addition, miRNA microarray analysis revealed the overexpression of miR‐92a‐3p in taurolithocholic acid 3‐sulfate‐treated AR42J cells.^16^ Zhang et al.^16^ also indicated that miR‐92a‐3p could inversely affect the activation of trypsinogen to mediate the progression of AP. However, the regulatory mechanism of miR‐92a‐3p in AP still need further investigation.

Protein inhibitor of activated STAT1 (Pias1) is a well‐studied E3 small ubiquitin‐like modifier ligases, which interacts with activated signal transducers and activators of transcription 1 (STAT1) to suppress its binding to DNA.^17^ Pias1 was reported to manage multiple cellular processes, including inflammation.^18^ In addition, silencing of Pias1 promoted inflammatory response and cell damage in caerulein‐stimulated pancreatic acinar cells.^19^ Chen et al.^20^ suggested that Pias1 negatively regulated STAT1 expression, thereby remitting the severity of SAP. Thus, Pias1 may be a promising target for curing AP, whereas the function and mechanism of Pias1 remains need in depth exploration.

In the current research, an in vitro AP model was established by stimulating MPC‐83 cells with caerulein. The expression levels of mmu_circ_0000037 in caerulein‐induced MPC‐83 cells were detected. Furthermore, functional experiments were performed to determine the function and regulatory mechanism of mmu_circ_0000037. This study aimed to seek the possible therapeutic biomarkers and provide theoretical support for AP treatment.

## 2 MATERIALS AND METHODS

### 2.1 Cell culture and treatment

The mouse pancreatic acinar cell line MPC‐83 was procured from BeNa Culture Collection and maintained in RPMI‐1640 medium (Thermo Fisher Scientific) containing 10% fetal bovine serum at 37°C with 5% CO_2_.

For establishment of AP cellular model, MPC‐83 cells were exposed to 10 nmol/L caerulein (Sigma) for 8 h and then the cells were collected for RNA or western blot analysis.^21^ For time‐dependent expression analysis, MPC‐83 cells were incubated with 10 nmol/L caerulein for 0, 4, 6, 8, and 10 h.

### 2.2 Cell transfection

pCD‐ciR overexpression vector containing mmu_circ_0000037 and empty vector (pCD‐ciR), miR‐92a‐3p mimic and inhibitor, small interfering RNA (siRNA) against Pias1 and their negative controls (NC mimic, NC inhibitor, and si‐NC) were procured from Genepharma. For cell transfection, MPC‐83 cells were plated into the six‐well plates until the cell density reached 60% and then the oligonucleotides were introduced into MPC‐83 cells using Lipofectamine^TM^ 2000 (Thermo Fisher Scientific).

### 2.3 MTT (3‐(4,5‐dimethylthiazol‐2‐yl)‐2,5‐diphenyltetrazolium bromide) assay

Briefly, MPC‐83 cells (1 × 10^4^/well) were seeded into the 96‐well plates and exposed to 10 nmol/L caerulein for 8 h. Twenty‐four hours upon incubation, cells were incubated with 0.5 mg/mL MTT solution (Beyotime) at 37°C for additional 4 h. Subsequently, 100 μL dimethyl sulfoxide (Sigma) was supplemented into each well for solubilization of formazan. The optical density value at 490 nm was estimated using a microplate reader (Bio‐Rad) and cell viability was determined by normalizing with matched control.

### 2.4 Enzymatic method measurement of amylase

MPC‐83 cells (1 × 10^7^) were harvested and resuspended in Amylase Assay Buffer, and then centrifuged at 1500*g* for 5 min at 4°C to collect supernatant. Cell samples were diluted to the appropriate concentration and then incubated with corresponding reagents in Amylase Assay Kit (Abcam). Then Amylase activity at 405 nm was assessed by a microplate reader (Bio‐Rad).

### 2.5 Enzyme‐linked immunosorbent assay (ELISA)

MPC‐83 cells (4 × 10^4^/well) were placed in 24‐well plates and then exposed to 10 nmol/L caerulein for 8 h. Subsequently, culture supernatant was harvested for the detection of the secretion of pro‐inflammatory cytokines tumor necrosis factor‐α (TNF‐α), interleukin 6 (IL‐6), interleukin 8 (IL‐8), and interleukin 1β (IL‐1β) using corresponding ELISA kit (Thermo Fisher Scientific).

### 2.6 Flow cytometry

MPC‐83 cells (5 × 10^5^/well) were cultured in 24‐well plates in triplicates and stimulated with 10 nmol/L caerulein for 8 h. After washing with phosphate‐buffered saline for three times, cells were resuspended and stained with Annexin V‐fluorescein isothiocyanate and propidium iodide (Beyotime) for 15 min. The cells were then estimated by a flow cytometer (BD) and apoptosis rate was defined as the sum of the percentage of the early and late apoptosis.

### 2.7 Quantitative real‐time polymerase chain reaction (RT‐qPCR)

RNA was isolated using Trizol reagent (Thermo Fisher Scientific). Then, 1 μg RNA was used to synthesize complementary DNA (cDNA) using All‐in‐One^TM^ miRNA First‐Strand cDNA Synthesis Kit (FulenGen) or Universal cDNA Synthesis Kit (Roche). The RT‐qPCR assay was conducted with SYBR mix (TaKaRa) and specific primers using Bio‐Rad CFX96 Real‐time PCR Systems (Bio‐Rad). The primer sequences were presented in Table 1. Relative expression levels of RNAs and mRNA were determined by 2^−ΔΔCt^ approach.^22^ Glyceraldehyde 3‐phosphate dehydrogenase (GAPDH) and U6 were used as endogenous controls.

**Table 1:** The primer sequences for RT‐qPCR assay.

Gene	Primer list (5′–3′)	
*mmu_circ_0000037*	Forward	TTGGGGTCTGTTCAGTCCTG
	Reverse	GCAACTTTATCATCAGCAGCC
*Mfsd6*	Forward	GCTCAGCCCTGAAACGGG
	Reverse	GCATGTCGGTTGGGTGGCTA
*mmu‐miR‐92a‐3p*	Forward	CGCGTATTGCACTTGTCCC
	Reverse	AGTGCAGGGTCCGAGGTATT
*Pias1*	Forward	GGACAGTGCGGAACTAAAGC
	Reverse	GCTGTGGAATGGTGGATGGA
*Gapdh*	Forward	CCCTTAAGAGGGATGCTGCC
	Reverse	ACTGTGCCGTTGAATTTGCC
*U6*	Forward	CTCGCTTCGGCAGCACAT
	Reverse	TTTGCGTGTCATCCTTGCG

Abbreviations: RT‐qPCR, quantitative real‐time polymerase chain reaction.

### 2.8 RNase R treatment and Actinomycin D (Act D) digestion assay

For RNase R treatment, total RNA (2 μg) was nurtured with or without RNase R (3 U/μg; Epicentre Technologies) at 37°C for 30 min, then the enrichment of mmu_circ_0000037 and Mfsd6 was assessed by RT‐qPCR.

For Act D treatment, MPC‐83 cells were incubated with 1 μM Act D for 0, 6, 12, and 24 h, respectively. Then, RNA was isolated at each time point and the expression of mmu_circ_0000037 and Mfsd6 was detected by RT‐qPCR assay.

### 2.9 Western blot analysis

The protein was isolated from cells using RIPA buffer (Beyotime) and quantified with BCA Kit (Beyotime). Subsequently, equal amounts of proteins were separated by sodium dodecyl‐sulfate polyacrylamide gel electrophoresis gel and then transferred onto polyvinylidene difluoride membranes (Millipore). After blocking with 5% skim milk for 1 h, the membranes were incubated at 4°C overnight with primary antibodies against Pias1 (ab32219, 1:2500 dilution, Abcam), B‐cell lymphoma‐2 (Bcl‐2)‐associated X protein (Bax) (ab182733, 1:1000 dilution, Abcam), Bcl‐2 (ab196495, 1:1000 dilution, Abcam), or cleaved caspase 3 (#9661, 1:1000 dilution, CST) and corresponding secondary antibody for 2 h at room temperature. GAPDH (ab181603, 1:10000 dilution, Abcam) was served as an inner control. The signals were visualized using EasyBlot ECL Kit (Sangon Biotech) and densitometry analysis was conducted with Quantity One software (Bio‐Rad).

### 2.10 Dual‐luciferase reporter assay

The binding sites between miR‐92a‐3p and Pias1 or mmu_circ_0000037 were forecasted by StarbaseV3.0 (http://www.sysu.edu.cn/). Then, mmu_circ_0000037 sequence or Pias1 3′‐untranslated region (3′UTR) sequence harbored the wild type (WT) or mutant type (MUT) miR‐92a‐3p‐binding sites were inserted into the pmirGLO vector (Promega) to generate the mmu_circ_0000037 WT, mmu_circ_0000037 MUT, Pias1‐WT, or Pias1‐MUT vectors. MPC‐83 cells were co‐introduced with miR‐92a‐3p mimic or miR‐NC and luciferase reporter vectors for 48 h using Lipofectamine^TM^ 2000. Then, luciferase activity was detected using dual‐luciferase reporter system (Promega).

### 2.11 RNA immunoprecipitation (RIP) assay

RIP assay was carried out to inspect the association between miR‐92a‐3p and mmu_circ_0000037 using the Magna RNA immunoprecipitation kit (Millipore). In brief, MPC‐83 cells introduced with miR‐92a‐3p mimic or miR‐NC were lysed in RIP lysis buffer. Then, cell lysates were cultured with anti‐Argonaute 2 antibody (Ago2; Millipore) or immunoglobulin G (Millipore) and adsorbed on the magnetic beads. The mixture was incubated with protease K to isolate immunoprecipitated RNA. The enrichment of mmu_circ_0000037 in each group was measured by RT‐qPCR.

### 2.12 Statistical analysis

Each experiment was repeated three times. Data of three replications were displayed as mean ± SD. Statistical analysis was managed by GraphPad Prism 7 software (GraphPad) and analyzed by Student's *t* test or one‐way analysis of variance followed by Tukey's test. *p* < .05 was deemed as significant difference.

## 3 RESULTS

### 3.1 Mmu_circ_0000037 is downregulated in caerulein‐induced MPC‐83 cells

**Figure 1:**
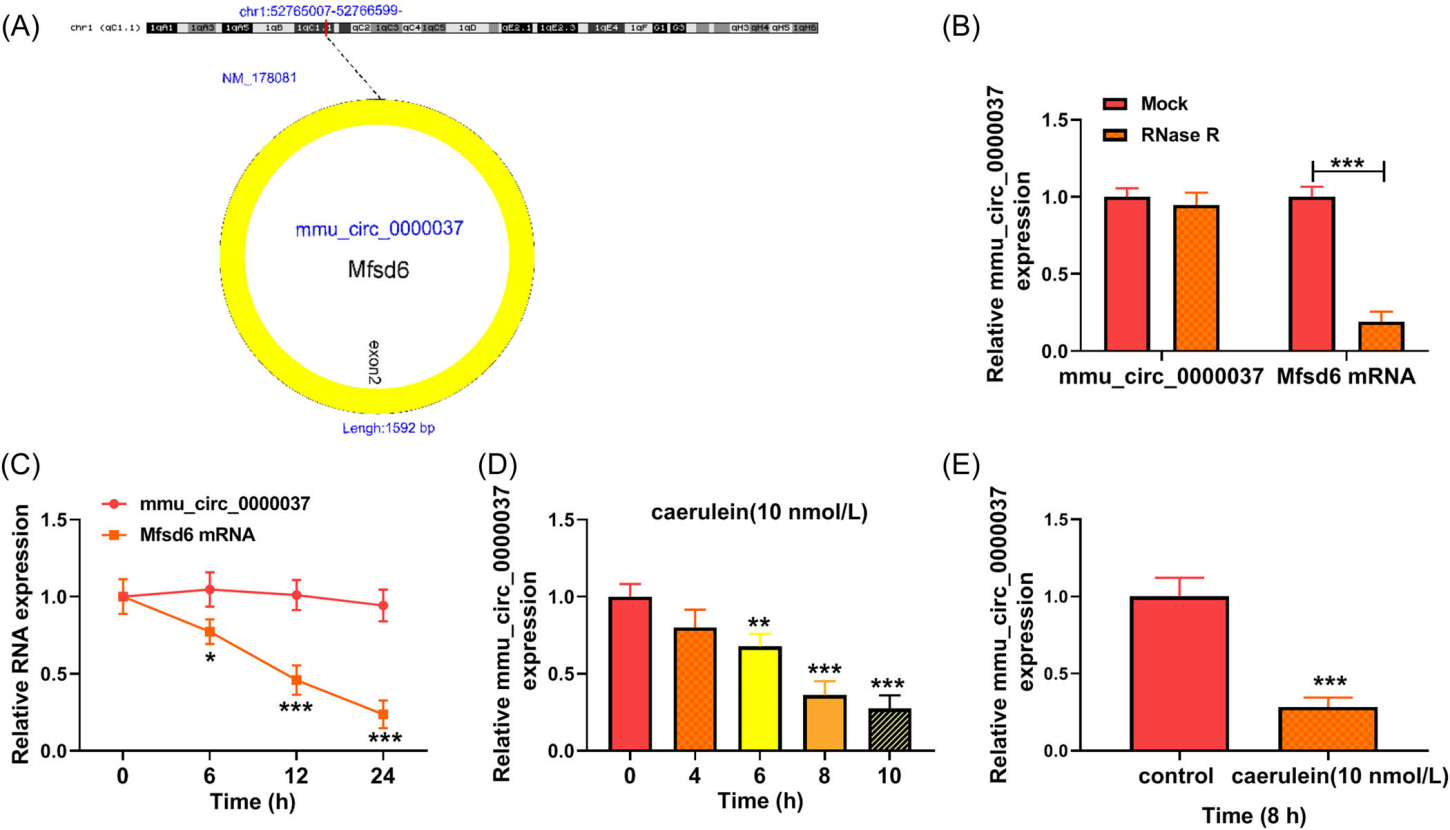
Mmu_circ_0000037 is lower expressed in caerulein‐induced MPC‐83 cells. (A) Schematic representation of the covalently closed circular structure of mmu_circ_0000037. (B) The expression levels of mmu_circ_0000037 and its linear transcript Mfsd6 messenger RNA (mRNA) in MPC‐83 cells with or without RNase R treatment were detected by quantitative real‐time polymerase chain reaction (RT‐qPCR). (C) The expression levels of mmu_circ_0000037 and its linear transcript Mfsd6 mRNA in MPC‐83 cells with actinomycin D treatment were detected by RT‐qPCR. (D) Relative expression level of mmu_circ_0000037 in MPC‐83 cells treated with 10 nmol/L caerulein for different times (0, 4, 6, 8, or 10 h) were detected by RT‐qPCR. (E) The mmu_circ_0000037 expression in MPC‐83 cells treated with 10 nmol/L caerulein for 8 h or nontreated (control) cells were detected by RT‐qPCR. *p < .05, **p < .01, ***p < .001.

According to the results of circRNA sequencing analysis, mmu_circ_0000037 is downregulated in the SAP mice compared with healthy controls.^9^ Nevertheless, the function and mechanism of mmu_circ_0000037 in AP has not been discussed. Mmu_circ_0000037 is located at chr1:52765007‐52766599, which derived from the *Mfsd6* gene (Figure 1A). To verify the circular structure of mmu_circ_0000037, RNase R and Act D digestion assays were performed. As shown in Figure 1B, RNase R treatment decreased Mfsd6 linear mRNA levels, whereas mmu_circ_0000037 was not digested. When cells were treated with Act D, the expression of mmu_circ_0000037 was not significantly decreased, while the expression of Mfsd6 was obviously decreased (Figure 1C). These results confirmed the circular structure of mmu_circ_0000037. To investigate the influence of mmu_circ_0000037 in AP, MPC‐83 cells were stimulated with 10 nmol/L caerulein for 0, 4, 6, 8, or 10 h to construct an in vitro cellular model for AP. The results indicated that mmu_circ_0000037 was significantly downregulated at 6, 8, or 10 h in MPC‐83 cells during caerulein treatment (Figure 1D). According to this results, MPC‐83 cells stimulated with 10 nmol/L caerulein for 8 h were selected for subsequent study. Besides, we compared the difference of mmu_circ_0000037 expression between the caerulein treatment group and the nontreated (control) group, and the results showed that mmu_circ_0000037 was significantly reduced in caerulein‐treated MPC‐83 cells (Figure 1E). These data indicated that mmu_circ_0000037 expression might be associated with AP progression.

### 3.2 Overexpression of mmu_circ_0000037 mitigates caerulein‐induced cell damage in MPC‐83 cells

**Figure 2:**
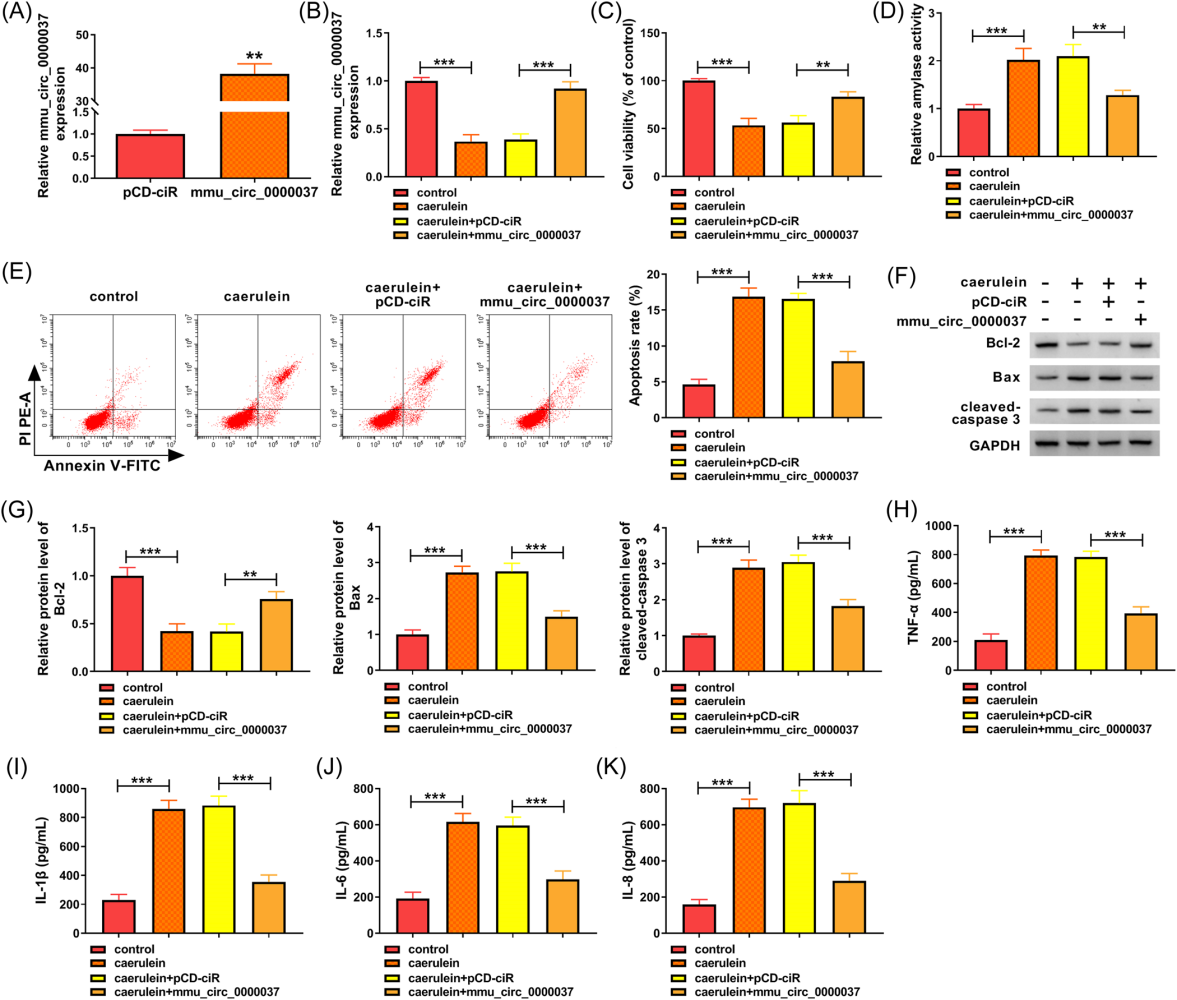
Mmu_circ_0000037 diminishes caerulein‐induced inflammatory injury in MPC‐83 cells. (A) The expression level of mmu_circ_0000037 in MPC‐83 cells transfected with pCD‐ciR or mmu_circ_0000037 overexpression vector. (B–K) MPC‐83 cells treated with or without 10 nmol/L caerulein for 8 h were transfected with pCD‐ciR or mmu_circ_0000037 overexpression vector. (B) The expression level of mmu_circ_0000037 in transfected cells was detected by quantitative real‐time polymerase chain reaction (RT‐qPCR). (C) Amylase activity in transfected cells. (E) The apoptosis rate of transfected cells was detected by flow cytometry assay. (F, G) The protein levels of B‐cell lymphoma‐2 (Bcl‐2), Bcl‐2‐associated X protein (Bax), and cleaved‐caspase‐3 in transfected cells were detected by western blot analysis. (H–K) Levels of tumor necrosis factor‐α (TNF‐α), interleukin 1β (IL‐1β), interleukin 6 (IL‐6), and interleukin 8 (IL‐8) were assessed by enzyme‐linked immunosorbent assay. **p < .01, ***p < .001.

To scrutinize the influence of mmu_circ_0000037 in AP, we overexpressed mmu_circ_0000037 in MPC‐83 cells and stimulated the cells with caerulein. RT‐qPCR assay indicated that mmu_circ_0000037 was nearly 40‐fold elevated by mmu_circ_0000037 overexpression vector in contrast to pCD‐ciR group (Figure 2A). Caerulein treatment significantly decreased the expression of mmu_circ_0000037, whereas this effect was partly overturned by overexpression of mmu_circ_0000037 (Figure 2B). Functional assay revealed that caerulein treatment suppressed cell viability (Figure 2C), but elevated the activity of amylase (Figure 2D) and apoptosis rate (Figure 2E). However, these effects were partly reversed by overexpression of mmu_circ_0000037 in caerulein‐induced MPC‐83 cells (Figure 2C–E). Western blot analysis revealed that mmu_circ_0000037 overexpression partly overturned caerulein‐induced the decline of Bcl‐2 and the elevation of Bax and cleaved‐caspase 3 (Figure 2F,G). Additionally, caerulein stimulation also facilitated the release of inflammatory cytokines in MPC‐83 cells, as identified by the extended levels of TNF‐α, IL‐1β, IL‐6, and IL‐8. However, mmu_circ_0000037 overexpression also overturned the effects of caerulein on the release of inflammatory cytokines in MPC‐83 cells (Figure 2H–K). Collectively, overexpression of mmu_circ_0000037 protects MPC‐83 cells from caerulein‐induced inflammatory injury.

### 3.3 Mmu_circ_0000037 acts as a sponge for miR‐92a‐3p

**Figure 3:**
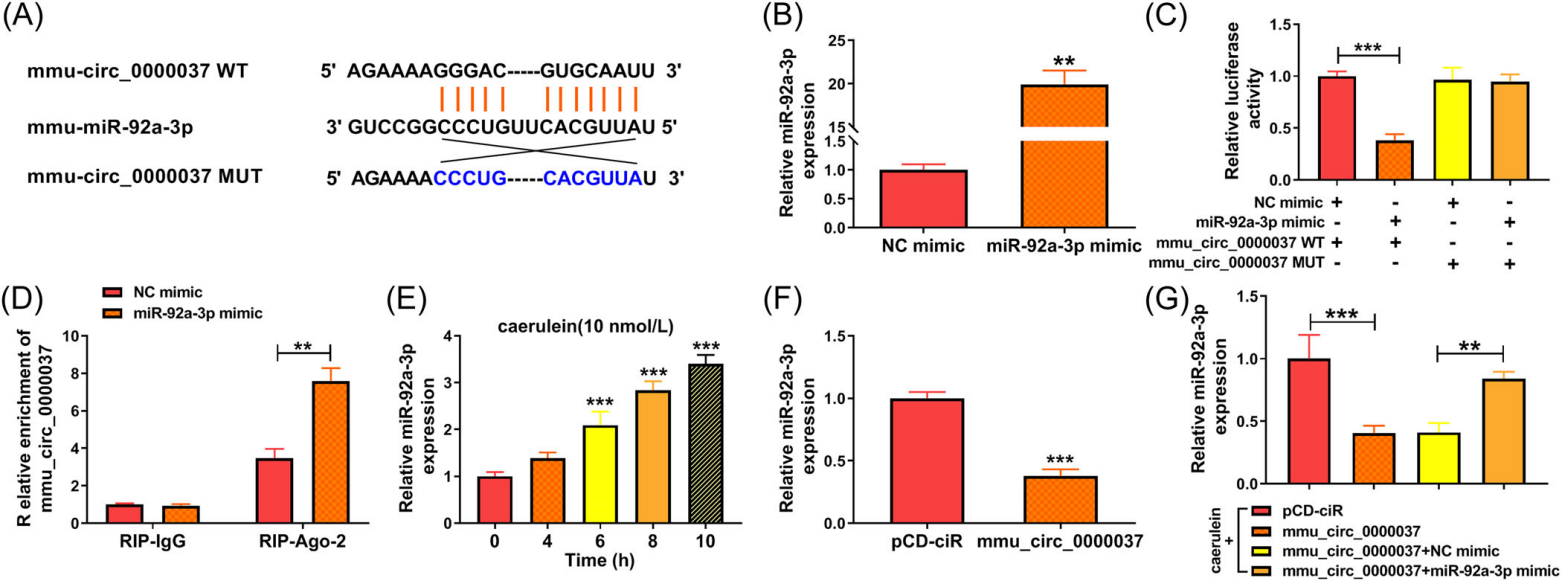
Mmu‐miR‐92a‐3p is targeted by mmu_circ_0000037. (A) Wild type (WT) or mutant type (MUT) binding sites between mmu_circ_0000037 and mmu‐miR‐92a‐3p were predicted by StarbaseV3.0. (B) The expression level of mmu‐miR‐92a‐3p in MPC‐83 cells transfected negative control (NC) mimic or miR‐92a‐3p mimic were detected by quantitative real‐time polymerase chain reaction (RT‐qPCR). (C) Dual‐luciferase reporter assay for the luciferase activity in MPC‐83 cells cotransfected with mmu_circ_0000037 WT or mmu_circ_0000037 MUT and NC mimic or miR‐92a‐3p mimic. (D) Argonaute 2 (Ago2) RNA immunoprecipitation (RIP) and RT‐qPCR assays for the enrichments of mmu_circ_0000037 in MPC‐83 cells transfected with NC mimic or miR‐92a‐3p mimic. (E) Relative expression level of mmu‐miR‐92a‐3p in MPC‐83 cells treated with 10 nmol/L caerulein for different times (0, 4, 6, 8, or 10 h) were detected by RT‐qPCR. (F) Expression level of mmu‐miR‐92a‐3p in MPC‐83 cells transfected with pCD‐ciR or mmu_circ_0000037 overexpression vector was detected by RT‐qPCR assay. (G) Expression level of mmu‐miR‐92a‐3p in MPC‐83 cells cotransfected with pCD‐ciR, mmu_circ_0000037, mmu_circ_0000037 + NC mimic or mmu_circ_0000037 + miR‐92a‐3p mimic. **p < .01, ***p < .001.

As circRNA regulated cell function by acting as miRNA sponges, we further forecasted the probable target miRNA of mmu_circ_0000037 by StarbaseV3.0. In addition, miR‐92a‐3p was predicted to be a target of mmu_circ_0000037. The complementary binding sites between mmu_circ_0000037 and miR‐92a‐3p are exhibited in Figure 3A. The expression of miR‐92a‐3p was elevated 20‐fold in MPC‐83 cells transfected with miR‐92a‐3p mimic (Figure 3B). In contrast with the NC mimic group, transfection of miR‐92a‐3p considerably decreased the luciferase activity of mmu_circ_0000037‐WT group, whereas it had little effect on the luciferase activity of mmu_circ_0000037‐MUT group (Figure 3C). Ago2‐RIP assay indicated that miR‐92a‐3p mimic increased the enrichment of mmu_circ_0000037 in Ago2‐immunoprecipitate group (Figure 3D). The expression of miR‐92a‐3p was elevated in caerulein‐stimulated MPC‐83 cells (Figure 3E), but it was downregulated by mmu_circ_0000037 overexpression (Figure 3F). Moreover, miR‐92a‐3p mimic partly overturned the suppression effect of mmu_circ_0000037 overexpression vector on miR‐92a‐3p expression (Figure 3G). Altogether, mmu_circ_0000037 acts as miR‐92a‐3p sponge to negatively regulate miR‐92a‐3p expression.

### 3.4 Mmu_circ_0000037 inhibits caerulein‐induced inflammatory injury in MPC‐83 cells by sponging miR‐92a‐3p

**Figure 4:**
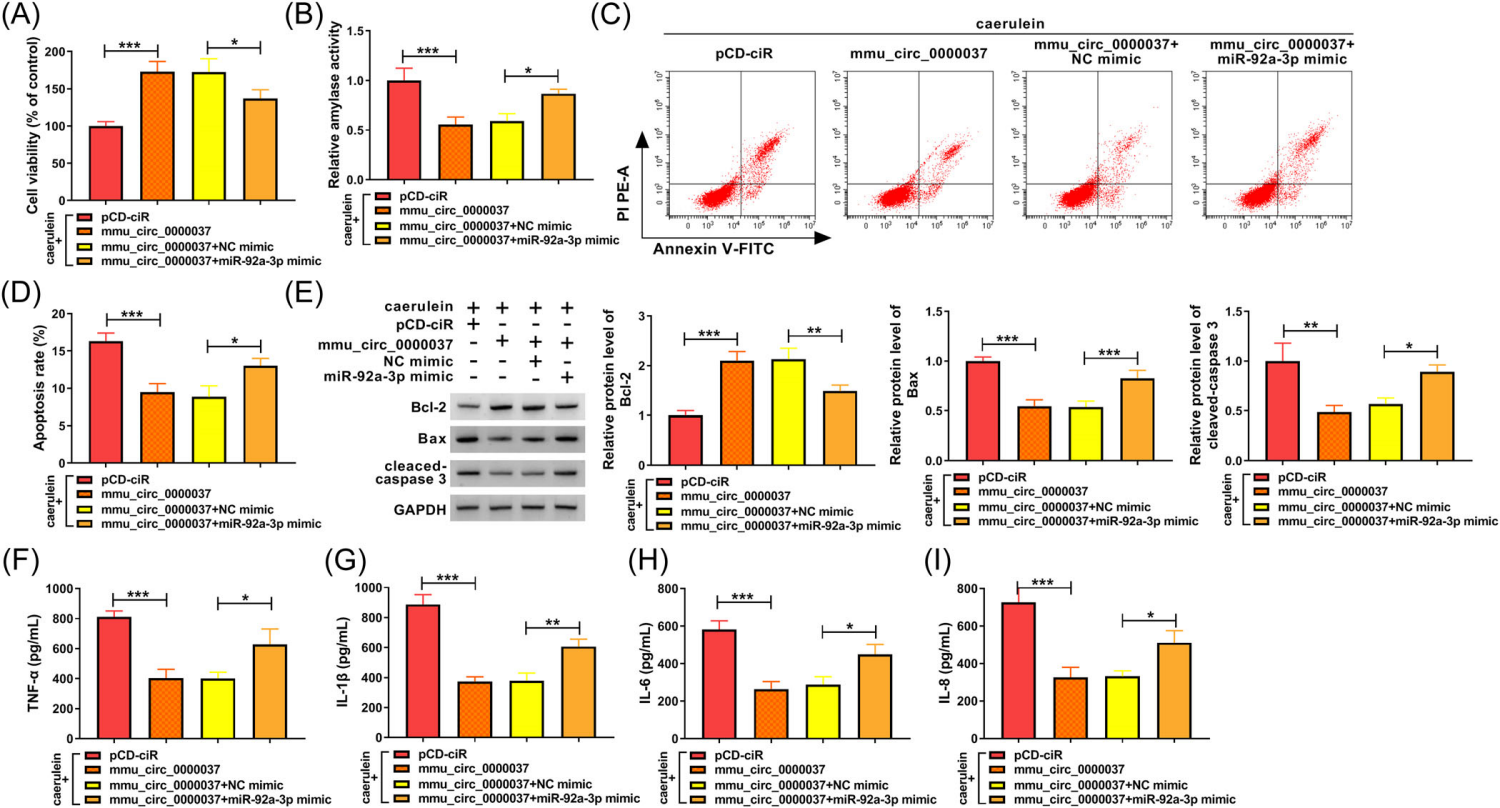
Overexpression of miR‐92a‐3p attenuates the function of mmu_circ_0000037 in caerulein‐induced MPC‐83 cells. (A–I) Caerulein‐induced MPC‐83 cells were transfected with pCD‐ciR, mmu_circ_0000037, mmu_circ_0000037 + NC mimic or mmu_circ_0000037 + miR‐92a‐3p mimic, respectively. Then, cell viability (A), Amylase activity (B), apoptosis rate (C, D), apoptosis‐related protein levels (E), and inflammatory cytokines levels (F–I) in transfected cells were determined. *p < .05, **p < .01, ***p < .001.

We further investigated the function of miR‐92a‐3p in caerulein‐induced MPC‐83 cells by loss‐of‐function experiments. Overexpression of miR‐92a‐3p remarkably overturned mmu_circ_0000037‐induced the promotion of cell viability (Figure 4A), the decrease of amylase activity (Figure 4B), apoptosis (Figure 4C‐E), as well as the inhibition of inflammatory factors secretion (Figure 4F–I) in caerulein‐induced MPC‐83 cells. These data uncovered that mmu_circ_0000037 could remit caerulein‐induced MPC‐83 cells by interacting with miR‐92a‐3p.

### 3.5 Pias1 is a target of miR‐92a‐3p in MPC‐83 cells

**Figure 5:**
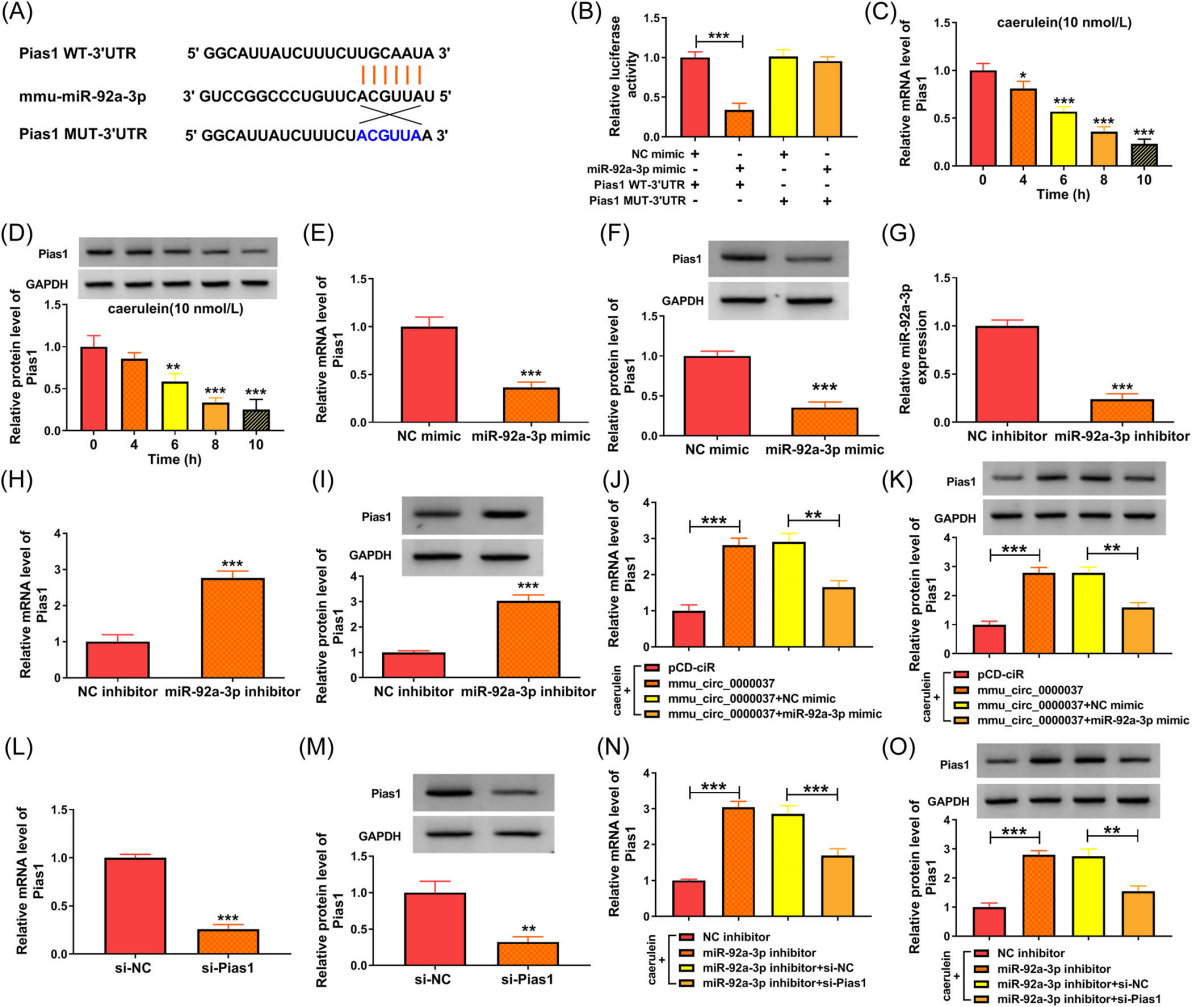
Protein inhibitor of activated STAT1 (Pias1) is a target of miR‐92a‐3p in MPC‐83 cells. (A) The complementary binding sites between miR‐92a‐3p and Pias1 were predicted by StarbaseV3.0. (B) Luciferase reporter assay was performed to confirm the correlation between miR‐92a‐3p and Pias1. (C, D) The messenger RNA (mRNA) and protein levels of Pias1 in MPC‐83 cells treated with 10 nmol/L caerulein for different times (0, 4, 6, 8, or 10 h) were detected by quantitative real‐time polymerase chain reaction (RT‐qPCR) or western blot analysis. (E, F) The mRNA and protein levels of Pias1 in MPC‐83 cells transfected with negative control (NC) mimic or miR‐92a‐3p mimic. (G) The expression level of miR‐92a‐3p in MPC‐83 cells transfected with NC inhibitor or miR‐92a‐3p inhibitor. (H, I) The mRNA and protein levels of Pias1 in MPC‐83 cells transfected with NC inhibitor or miR‐92a‐3p inhibitor. (J, K) The mRNA and protein levels of Pias1 in caerulein‐induced MPC‐83 cells transfected with pCD‐ciR, mmu_circ_0000037, mmu_circ_0000037 + NC mimic, or mmu_circ_0000037 + miR‐92a‐3p mimic. (L, M) RT‐qPCR assay for the mRNA and protein levels of Pias1 in MPC‐83 cells transfected with si‐NC or si‐Pias1. (N, O) The mRNA and protein levels of Pias1 in caerulein ‐induced MPC‐83 cells transfected with NC inhibitor, miR‐92a‐3p inhibitor, miR‐92a‐3p inhibitor + si‐NC, or miR‐92a‐3p inhibitor + si‐Pias1. *p < .05, **p < .01, ***p < .001.

We then predicted the potential targets of miR‐92a‐3p by using StarbaseV3.0 database and the complementary miR‐92a‐3p‐binding fragments were found in the 3′UTR of Pias1 sequence. Then, Pias1 sequence harbored the WT‐ or MUT‐type miR‐92a‐3p‐binding motifs were inserted into the dual‐luciferase reporter vectors to confirm the interaction between them (Figure 5A). As presented in Figure 5B, high expression of miR‐92a‐3p diminished the luciferase activity of Pias1 WT‐3′UTR group in contrast with transfection of NC mimic, whereas had no significant influence on the luciferase activity of Pias1 MUT‐3′UTR group. Besides, we further investigated Pias1 expression in caerulein‐induced MPC‐83 cells. As shown in Figure 5C,D, Pias1 mRNA and protein levels were decreased with time of caerulein treatment. Additionally, the mRNA and protein levels of Pias1 in MPC‐83 cells was remarkably lowered by miR‐92a‐3p overexpression (Figures 5E,F). After confirmation that miR‐92a‐3p inhibitor indeed reduced miR‐92a‐3p expression (Figure 5G), we detected Pias1 expression and confirmed that the mRNA and protein levels of Pias1 were elevated by miR‐92a‐3p inhibitor (Figures 5H,I). Meanwhile, mmu_circ_0000037 overexpression significantly elevated Pias1 at mRNA and protein levels, whereas miR‐92a‐3p partly overturned this effect in caerulein‐induced MPC‐83 cells (Figure 5J,K). The performance maintained that mmu_circ_0000037 upregulated Pias1 expression by absorbing miR‐92a‐3p. To determine if miR‐92a‐3p acted as a sponge of Pias1, siRNA against Pias1 was constructed and functional rescue experiment was conducted. As displayed in Figure 5L,M, Pias1 levels were decreased more than half in cells with si‐Pias1 transfection, suggesting a successful knockdown of Pias1. In addition, Pias1 mRNA and protein levels in caerulein‐induced MPC‐83 cells were elevated by miR‐92a‐3p downregulation, while these effects were partly overturned by Pias1 knockdown (Figure 5N,O). Taken together, mmu_circ_0000037 modulated Pias1 expression via performing as a ceRNA of miR‐92a‐3p.

### 3.6 Pias1 contributes to the regulation of miR‐92a‐3p on caerulein‐induced inflammatory injury in MPC‐83 cells

**Figure 6:**
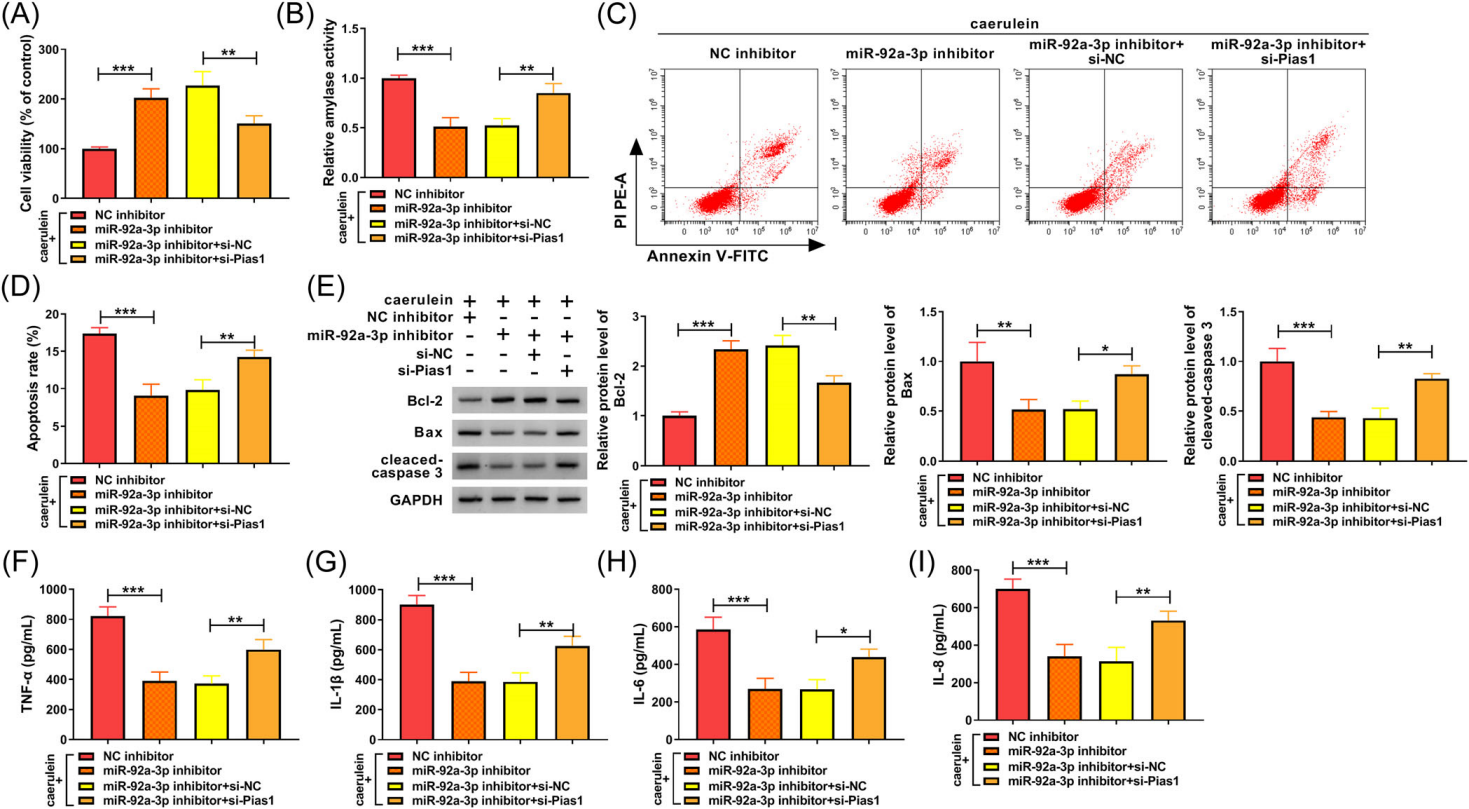
Protein inhibitor of activated STAT1 (Pias1) restoration reverses the effects of miR‐92a‐3p on caerulein‐induced MPC‐83 cells. Cell viability (A), Amylase activity (B), apoptosis rate (C, D), apoptosis‐related protein levels (E), and inflammatory cytokines levels (F–I) in MPC‐83 cells transfected with negative control (NC) inhibitor, miR‐92a‐3p inhibitor, miR‐92a‐3p inhibitor + si‐NC, or miR‐92a‐3p inhibitor + si‐Pias1 were determined. *p < .05, **p < .01, ***p < .001.

To explore whether miR‐92a‐3p mediated caerulein‐induced MPC‐83 cell injury by targeting Pias1, MPC‐83 cells were introduced with miR‐NC, miR‐92a‐3p inhibitor, miR‐92a‐3p inhibitor + si‐NC or miR‐92a‐3p inhibitor + si‐Pias1, and then stimulated by caerulein. As displayed in Figure 6A, downregulation of miR‐92a‐3p induced the increase of cell viability in caerulein‐induced MPC‐83 cells was markedly counteracted by Pias1 knockdown. Besides, knockdown of Pias1 weakened the restrictive effect of miR‐92a‐3p inhibitor on amylase activity (Figure 6B), apoptosis (Figure 6C–E), and inflammation (Figure 6F–I) in caerulein‐induced MPC‐83 cells. These results implied that miR‐92a‐3p encouraged caerulein‐induced inflammatory injury in MPC‐83 cells by decreasing Pias1.

## 4 DISCUSSION

Due to the limited understanding of AP pathophysiology, the treatment of this disease remains not specific. Hence, it is vitally important to investigate the mechanism underlying AP to find novel therapeutic strategy. CircRNAs have reported to perform important roles in many cell biology processes, including cell proliferation, apoptosis, and inflammation.^23^, ^24^ In current research, MPC‐83 cells were exposed to caerulein to establish the in vitro cell model for AP. The expression levels of mmu_circ_0000037 and Pias1 were diminished, whereas miR‐92a‐3p was elevated in caerulein‐stimulated MPC‐83 cells. Besides, gain‐ or loss‐of‐function assays were carried out to verify the function of these RNAs and gene in caerulein‐induced MPC‐83 cells. In addition, the modulation network among mmu_circ_0000037, miR‐92a‐3p, and Pias1 was constructed by functional recovery experiments. Our study provided clues to reveal the pathogenesis of AP.

Recent research disclosed that severe pathological changes in pancreatic acinar cells occupy a prominent place in AP occurrence and development.^25^ Apoptosis is a common programmed cell death, which has a significant influence during the course of AP.^26^, ^27^ Bcl‐2 family controls the intrinsic apoptotic pathway,^28^ in which Bcl‐2 protein is associated with antiapoptotic response and Bax mediates proapoptotic process by leading to irreparable damage to mitochondria.^29^, ^30^ Moreover, caspase 3 is also a main mediator of apoptosis and its activation (c‐caspase 3) is essential for apoptotic progression.^31^ Inflammatory response is another feature of AP,^32^ and some inflammatory cytokines such as TNF‐α, IL‐6, and IL‐1β are vital to inflammatory diseases.^33^ In addition, the increased amylase and lipase activity are another leading course of AP.^34^ Functional experiments revealed that stimulation of caerulein induced MPC‐83 cell apoptosis, increased amylase activity, and promoted the release of inflammatory factors, suggesting the successful establishment of AP model in vitro.

Mmu_circ_0000037 is a newly discovered circRNA, which was reported to be lower expressed in the pancreatic tissues from the mice with SAP.^9^ In accordance with previous research, our data showed that mmu_circ_0000037 was lower expressed in caerulein‐induced MPC‐83 cells. Besides, we confirmed that mmu_circ_0000037 overexpression significantly relieved caerulein‐stimulated cell injury by regulating cell viability, amylase activity, apoptosis, and inflammation. The above data showed that mmu_circ_0000037 could decrease amylase activity, apoptosis, and inflammation of caerulein‐stimulated MPC‐83 cells, which might slow down AP progression.

It is widely known that circRNAs act as miRNA sponges to regulate miRNA levels.^10^ In the present research, miR‐92a‐3p is targeted by mmu_circ_0000037. Their correlation was further affirmed by dual‐luciferase activity and RIP assay, as identified by the decreased luciferase activity of miR‐92a‐3p mimic and mmu_circ_0000037 WT cotransfected group, as well as the enrichment of mmu_circ_0000037 in Ago2 group. Besides, we pointed out that mmu_circ_0000037 negatively regulated the level of miR‐92a‐3p in MPC‐83 cells and overexpression of miR‐92a‐3p partly overturned the inhibition effect of mmu_circ_0000037 on caerulein‐stimulated MPC‐83 cell amylase activity, apoptosis, and inflammation. Moreover, miR‐92a‐3p inhibitor had been verified to repress caerulein‐induced MPC‐83 cell injury. Thus, we confirmed that mmu_circ_0000037 sponged miR‐92a‐3p to inhibit AP progression.

For investigation of miR‐92a‐3p promising target in AP progression, we performed bioinformatics analysis and confirmed that Pias1 contained the complementary binding sites of miR‐92a‐3p in 3′UTR sequence. Pias1, a transcriptional coregulator that selectively mediates STAT1‐dependent gene expression, exhibits a prominent preference for inflammatory response.^17^ Previous study showed that downregulation of Pias1 could enhance the expression of inflammatory mediators and induce cell injury in AP.^19^, ^20^ These data provided evidence that Pias1 might be a potential therapeutic target for AP. In our research, we found that miR‐92a‐3p could target Pias1 and Pias1 was downregulated in caerulein‐stimulated MPC‐83 cells. In addition, rescue experiments revealed that miR‐92a‐3p inhibitor inhibited caerulein‐induced AP cell injury by targeting Pias1 in vitro. Importantly, we pointed out that mmu_circ_0000037 could upregulate Pias1 expression by sponging miR‐92a‐3p, which improved the conclusion that mmu_circ_0000037 sponged miR‐92a‐3p to regulate Pias1, thereby mediating the progression of AP.

**Figure 7:**
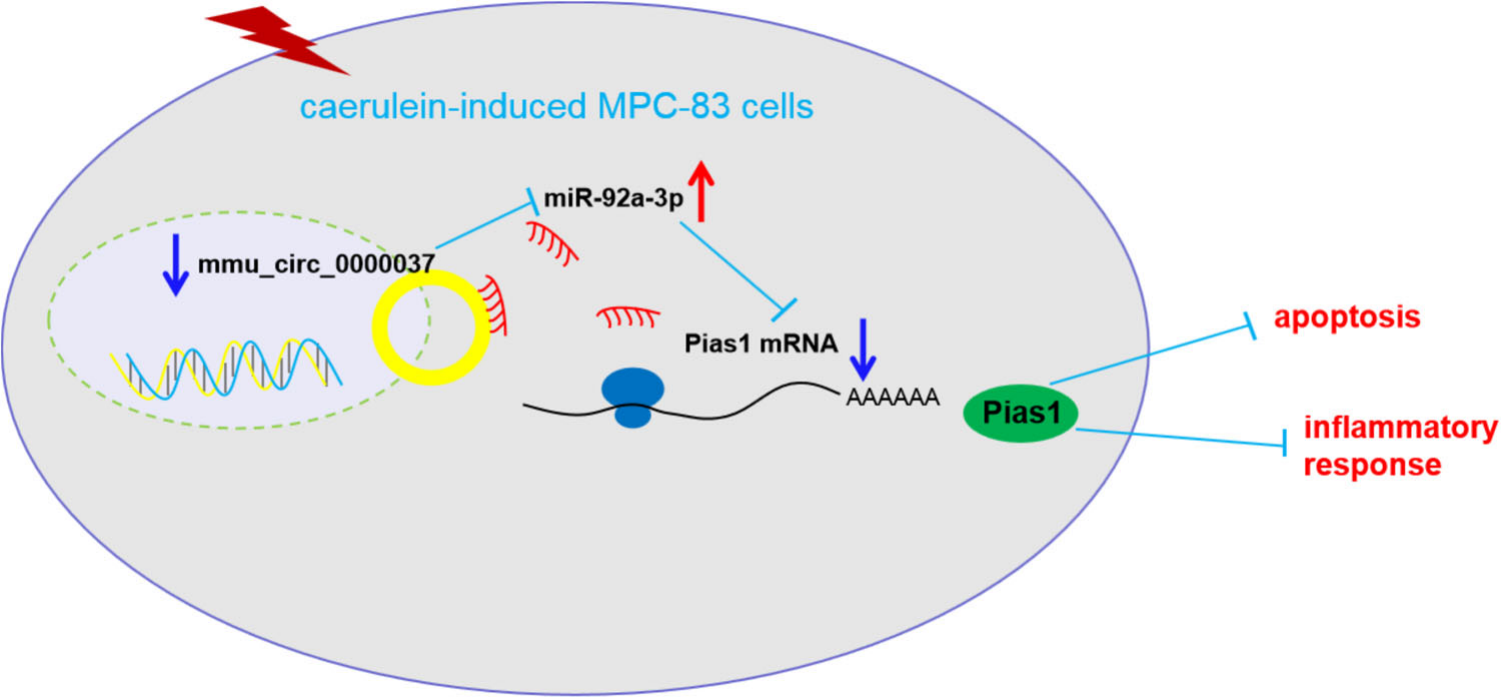
Mmu_circ_0000037 regulated cell apoptosis and inflammatory response by sponging miR‐92a‐3p to deregulate the regulation on miR‐92a‐3p target protein inhibitor of activated STAT1 (Pias1). mRNA, messenger RNA.

In conclusion, our research reveals for the first time the expression and function of mmu_circ_0000037 in caerulein‐induced MPC‐83 cells. Our data showed that mmu_circ_0000037 participated in the pathogenesis of AP, which could inhibit the apoptosis and inflammatory response of caerulein‐stimulated MPC‐83 cells by sponging miR‐92a‐3p to regulate Pias1 expression (Figure 7). Our results enhance the understanding of AP pathogenesis and provide the potential therapeutic targets for AP.

## AUTHOR CONTRIBUTIONS

Hua Chen and Jun Tu performed the research. Lei He, Ning Gao, and Weiqiang Yang designed the research study. Lei He, Ning Gao, and Weiqiang Yang contributed essential reagents or tools. Hua Chen and Jun Tu analyzed the data. Hua Chen and Jun Tu wrote the paper.

## CONFLICT OF INTEREST STATEMENT

The authors declare no conflicts of interest.
